# Psychosocial contributors to Internet and social media addiction among adult women

**DOI:** 10.1192/j.eurpsy.2022.2118

**Published:** 2022-09-01

**Authors:** K. Rachubińska, A. Cybulska, M. Szkup, D. Schneider-Matyka, E. Grochans

**Affiliations:** 1 Pomeranian Medical University in Szczecin, Department Of Nursing, Szczecin, Poland; 2 Pomeranian Medical University, Department Of Nursing, Szczecin, Poland

**Keywords:** internet addiction, behaviora addiction, women

## Abstract

**Introduction:**

When speaking of behavioral addictions (especially to the Internet and social media), it is emphasized that it is not the environment that is the main contributor to addiction, but rather certain behaviors and personality traits.

**Objectives:**

The aim of this study was to assess the level of Internet and social media addiction on the example of Facebook with regard to psychological and social factors.

**Methods:**

This survey-based study involved a group of women representing the female population in the West Pomeranian Voivodeship, Poland (N = 556). Research instruments were a self-developed questionnaire concerning sociodemographic data, the De Jong Gierveld Loneliness Scale, the Beck Depression Inventory, the Internet Addiction Test, and the Bergen Facebook Addiction Scale.

**Results:**

Age, depressive symptoms, loneliness were the variable contributing to Internet and Facebook addiction among the studied. Available studies confirm the results of their own research.

**Table:** 

	Employed n = 496	Unemployed n = 60	p
BDI	4.0(1.0 – 10.0)	6.5 (1.5 – 12.5)	0.20
*DJGLS*	34.1 ± 3.7	33.0 ± 5.0	0.09
IAT	32.0 (24.0 – 44.0)	24.5 (20.0 – 32.0)	< 0.001
BFAS	8.0 (6.0 – 12.0)	6.0 (6.0 – 7.5)	< 0.001

**Conclusions:**

Depressive symptoms and dependence on the Internet and Facebook were more common among single women. In the employed women, we only observed higher levels of Internet and Facebook addiction. The level of dependence on the Internet and Facebook was higher among younger women. Loneliness correlated with Internet and Facebook addiction, and more severe depressive symptoms entailed higher levels of Internet and Facebook addiction.

**Disclosure:**

No significant relationships.

